# Circadian Rhythm Regulates Reactive Oxygen Species Production and Inhibits Al-Induced Programmed Cell Death in Peanut

**DOI:** 10.3390/life12081271

**Published:** 2022-08-19

**Authors:** Aaron Ntambiyukuri, Xia Li, Dong Xiao, Aiqin Wang, Jie Zhan, Longfei He

**Affiliations:** 1 National Demonstration Center for Experimental Plant Science Education, College of Agriculture, Guangxi University, Nanning 530004, China; 2 Guangxi Key Laboratory for Agro-Environment and Agro-Product Safety, Nanning 530004, China; 3 Guangxi Colleges and Universities Key Laboratory of Crop Cultivation and Tillage, Nanning 530004, China

**Keywords:** circadian clock, reactive oxygen species, Al-induced PCD, photoperiodism, peanut

## Abstract

Peanut is among the most important oil crops in the world. In the southern part of China, peanut is highly produced; however, the arable land is acidic. In acidic soils, aluminum (Al) inhibits plant growth and development by changing the properties of the cell wall and causing the disorder of the intracellular metabolic process. Circadian rhythm is an internal mechanism that occurs about every 24 h and enables plants to maintain internal biological processes with a daily cycle. To investigate the effect of photoperiod and Al stress on the Al-induced programmed cell death (PCD), two peanut varieties were treated with 100 μM AlCl_3_ under three photoperiodic conditions (8/16, SD; 12/12, ND; 16/8 h, LD). The results show that Al toxicity was higher in ZH2 than in 99-1507 and higher under LD than under SD. Root length decreased by 30, 37.5, and 50% in ZH2 and decreased by 26.08, 34.78, and 47.82% in 99-1507 under SD, ND, and LD, respectively, under Al stress. Photoperiod and Al induced cell death and ROS production. MDA content, PME activity, and LOX activity increased under SD, ND, and LD, respectively, under Al stress both in ZH2 and 99-1507. APX, SOD, CAT, and POD activities were higher under SD, ND, and LD, respectively. Al stress increased the level of *AhLHY* expression under SD and ND but decreased it under LD in both ZH2 and 99-1507. Contrastingly, *AhSTS* expression levels increased exponentially and were higher under SD, LD, and ND, respectively, under Al stress. Our results will be a useful platform to research PCD induced by Al and gain new insights into the genetic manipulation of the circadian clock for plant stress response.

## 1. Introduction

It has been shown that about 50% of the total arable land around the world is acidic [1]. Al can be concentrated in the plant root tips and interfere with areas of plant growth, such as root and shoot growth, decrease biomass production and nutrient imbalance, and alter physiological and metabolic parameters, which will lead to a decrease in crop yield [2]. The presence of Al in the environment affects plants in various ways, including the generation of ROS [3]. Numerous reports have revealed mechanisms such as Al exclusion from the roots and evacuation to the vacuole in response to Al toxicity in rice [4,5], wheat, barley, maize [6,7], and rye and Arabidopsis [8]. QTLs related to Al tolerance have been identified and used to develop Al-tolerant crops such as maize [9,10], Arabidopsis [11], wheat [12], and rice [13] using breeding or molecular approaches. Genes such as MATE, ALMT, ASR, and ABC transporters have been implicated in some plants for resistance to Al [14], for instance, Al-responsive genes in potato [15], rice [16], wheat [17], sorghum [18], rye [19], and sugarcane [20]. Various transcription factors induced by Al stress, such as STOP1 [21,22], STOP2 [23], ART1, ASR5, and STAR1 [24], regulating the other Al-responsive genes to confer Al tolerance, have been reported. There are less reports on the influence of photoperiod on Al stress tolerance in plants; therefore, we need to understand how day length affects Al stress resistance in peanut. 

Circadian clock is an intrinsic timekeeping mechanism that synchronizes with the periodic environment through daily entrainment, especially light and temperature to adjust the internal rhythm [25,26,27]. The circadian clock anticipates daily environmental fluctuations by coordinating diverse physiological and developmental processes in a day-specific manner to enhance plant fitness and survival [28,29,30]. The circadian clock plays an important role under adverse environmental conditions, modulating biotic and abiotic stress responses [31,32]. However, many aspects, such as circadian rhythm and its role in Al stress, remain unclear. 

Programmed cell death (PCD) or apoptosis is a molecular process in which cells that are not needed commit suicide by activating an intracellular death program [33]. Numerous reports suggested that one of the mechanisms of PCD is the elimination of specific cells under developmental or environmental stimuli such as an increase in ROS in the presence of abiotic stress [34,35,36]. Photoperiod length is detected by a sensing mechanism consisting of chloroplasts and photoreceptors, which transfer the light information to the circadian clock. This photoperiod sensing influences the development of plants, induces abiotic and biotic stress tolerance, and causes photoperiod stress [37]. It has been revealed that short-day entrained plants were more affected than long-day entrained plants, and the minimal light treatment of 12 h could be necessary for inducing PCD [38]. The circadian clock contributes to cellular processes that maintain ROS at physiological levels in diverse organisms such as mouse [39], zebrafish [40], and *Arabidopsis thaliana* [41]. Plants under the long-day period exhibit reduced CCA1/LHY expression and the induction of PCD. Contrastingly, there was no PCD observed after short nights, so the expression of CCA1/LHY was similar to wild-type levels or higher [42], and it has been reported that CCA1 is a key regulator of ROS homeostasis through association with the evening component (EC) in ROS promoter genes [43]. The proper matching of internal circadian timing with environment enhances plant fitness and survival [27]. Circadian stress (perturbation) regimes have detrimental consequences that lead to the failure of ROS removal and induced PCD. However, the regulatory role of the circadian clock and day length on ROS production and the regulation of Al-induced PCD remains unclear. 

Antioxidant defense mechanisms keep the formed ROS at a low level [44,45,46,47]. CAT is the first peroxisomal antioxidant enzyme that detoxifies cellular H_2_O_2_ to be characterized [48], and its expression in Arabidopsis is controlled by the circadian clock [41]. It has been revealed that APX and CAT enzymes are involved in the elimination of H_2_O_2_ and ROS scavenging in the roots of rice [49]. There is no available report on the exact mechanism of the circadian clock regulating Al-induced PCD by regulating the antioxidant system under photoperiod and Al stress. To further understand the contribution of the circadian clock in the regulation of Al-induced PCD, we investigated the effect of different photoperiods and Al stress on root growth, membrane lipid peroxidation, ROS production, enzyme activities, and gene expression in peanuts.

## 2. Results

### 2.1. Effects of Photoperiod and Al Stress on Root Elongation and Cell Death 

**Figure 1:**
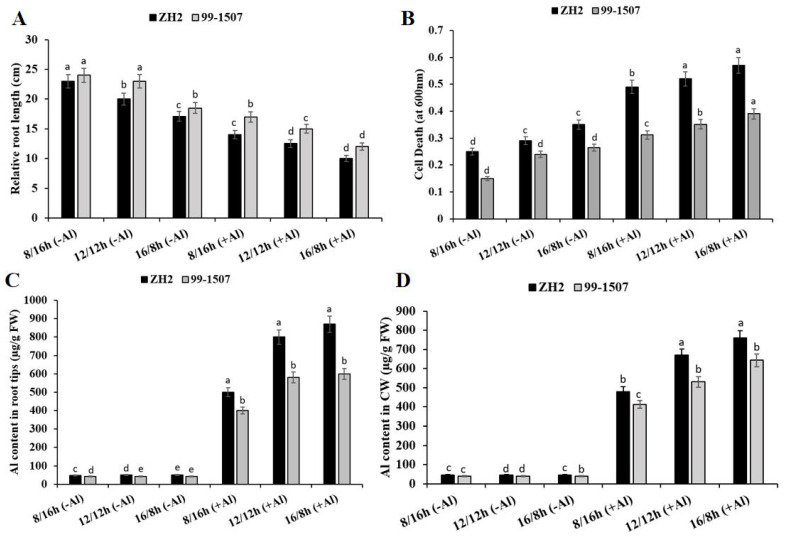
Effect of photoperiodism and Al stress on the root tips in peanut. (**A**) Relative root length (cm); (**B**) Root tip cell death; (**C**) Al content in the root tips; (**D**) Al content in the cell wall. Peanut cultivars (Zh2 and 99-1507) were treated with 100 μM Al, different photoperiods: SD (short day, 8/16 h), ND (normal day, 12/12 h), and LD (long day, 16/8 h). The experiment was carried out in triplicate. The data are presented as means. All the samples were used as fresh weight. Different letters (a–d) assigned to the error bar represent different levels of significance. The results were significant at *p* < 0.05 with different letters.

Root growth and cell death were influenced by the interaction of photoperiod and Al stress. Maximum root growth was observed in 99-1507 without Al treatment under SD (short day, 12/12 h) (Figure 1A). Compared to ND, root growth increased by 15% but decreased by 14.5% in ZH2 and increased by 4.34% but decreased by 19.56% in 99-1507 under SD (short day, 8/16 h) and LD (long day, 16/8 h), respectively, without Al treatment. When treated with Al, compared to ND with no Al, root length decreased by 30, 37.5, and 50% in ZH2 and decreased by 26.08, 34.78, and 47.82% in 99-1507 under SD, ND, and LD. Al highly inhibited root growth under LD compared to SD and ND in ZH2 and 99-1507 (Figure 1A). Photoperiod also significantly influenced Al-induced cell death in the root tips of peanut (Figure 1B). Dead cells decreased by 13.79% but increased by 20.68% in ZH2 and decreased by 37.5% but increased by 10.41% in 99-1507 under SD and LD, respectively, without Al treatment. Compared to ND with no Al, death cells increased by 68.96, 79.3, and 96.55% in ZH2 and increased by 30, 46.25, and 62.5% in 99-1507 under SD, ND, and LD, respectively, under Al stress. The inhibition of root growth and cell death induced by Al stress were higher under LD than ND and SD (Figure 1A,B). 

### 2.2. Effects of Photoperiod and Al Stress on Al Accumulation in the Root Tips and CW 

The treatment of ZH2 and 99-1507 under ND, SD, and LD periods with or without Al stress showed that Al content in root tips was highly observed in ZH2 and 99-1507 under LD and SD compared to ND (Figure 1C). However, there was no significant change in Al content in the root tips of ZH2 and 99-1507 under SD, ND, and LD, respectively, without Al treatment (Figure 1C). However, after Al treatment, Al content in the root tips increased exponentially by 10, 16, and 17.4 times in ZH2 under SD, ND, and LD, respectively, and increased by 9.52, 13.8, and 14.28 times in 99-1507 under SD, ND, and LD, respectively. There was no significant change in the Al content observed in CW without Al treatment (Figure 1D). However, with Al treatment, compared to ND with no Al, Al content was 10.66, 14.88, and 16.88 times higher in ZH2 under SD, ND, and LD, respectively, and 10.3, 13.25, and 16.075 times higher in 99-1507 under SD, ND, and LD, respectively. There was a very big difference between the influence of photoperiod alone and photoperiod with Al stress on the content of Al in the root tips and CW, and this was highly observed in ZH2 and 99-1507 under LD and SD when compared to the ND (Figure 1C,D).

### 2.3. Effects of Photoperiod and Al Stress on MDA Content, PME Activity, and LOX Activity

**Figure 2:**
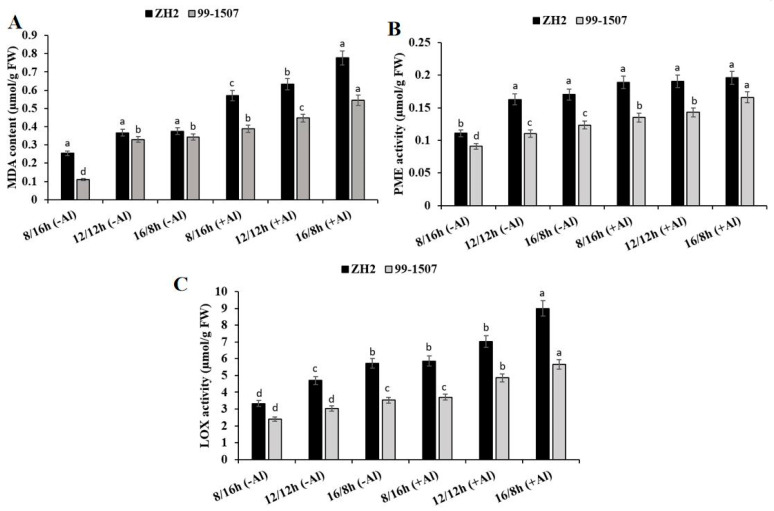
Effect of photoperiodism and Al on MDA content, PME, and LOX activities. (**A**) MDA content; (**B**) PME activity; and (**C**) LOX activity. The root tips of ZH2 and 99-1507 were treated with 100 µM Al under different light/dark periods for 24 h. The experiment was carried out in triplicate to ensure significant results. The data are presented in mean ± standard deviation. All the samples were used as fresh weight. Different letters (a–d) assigned to the error bar represent different levels of significance. The results were significant at *p* < 0.05 with different letters.

Photoperiod influenced lipid peroxidation and induced MDA content in the root tips of ZH2 and 99-1507. However, this MDA content was highly increased under photoperiod and Al stress (Figure 2A). Compared to ND, without Al treatment, MDA content decreased by 30.79% but increased by 2.17% in ZH2 and decreased by 66.51% but increased by 4.24% in 99-1507 under SD and LD, respectively. After Al treatment, compared to ND with no Al, MDA content increased by 55.58, 72.75, and 111.71% in ZH2 and increased by 17.57, 35.45, and 65.15% in 99-1507 under SD, ND, and LD, respectively. PME activity was influenced by photoperiod and Al stress (Figure 2B). Compared to ND, without Al treatment, PME activity decreased by 31.96% but increased by 4.53% in ZH2 and decreased by 17.57% but increased by 11.86% in 99-1507 under SD and LD, respectively. Compared to ND with no Al, PME activity increased by 15.95, 17.17, and 20.24% in ZH2 and increased by 22.28, 29.89, and 50.36% in 99-1507 under SD, ND, and LD, respectively, under Al stress. From Figure 2C, LOX activity was induced by photoperiod and Al stress. Without Al treatment, LOX activity decreased by 29.2% but increased by 21.63% in ZH2 and decreased by 20.56% but increased by 16.89% in 99-1507. On the other side, LOX activity increased by 24.64, 49.5, and 91.26% in ZH2 and increased by 22.75, 60.74, and 87.5% in 99-1507 under SD, ND, and LD, respectively, under Al stress. 

### 2.4. Effects of Photoperiod and Al on ROS Production and Antioxidant Enzyme Activities 

**Figure 3:**
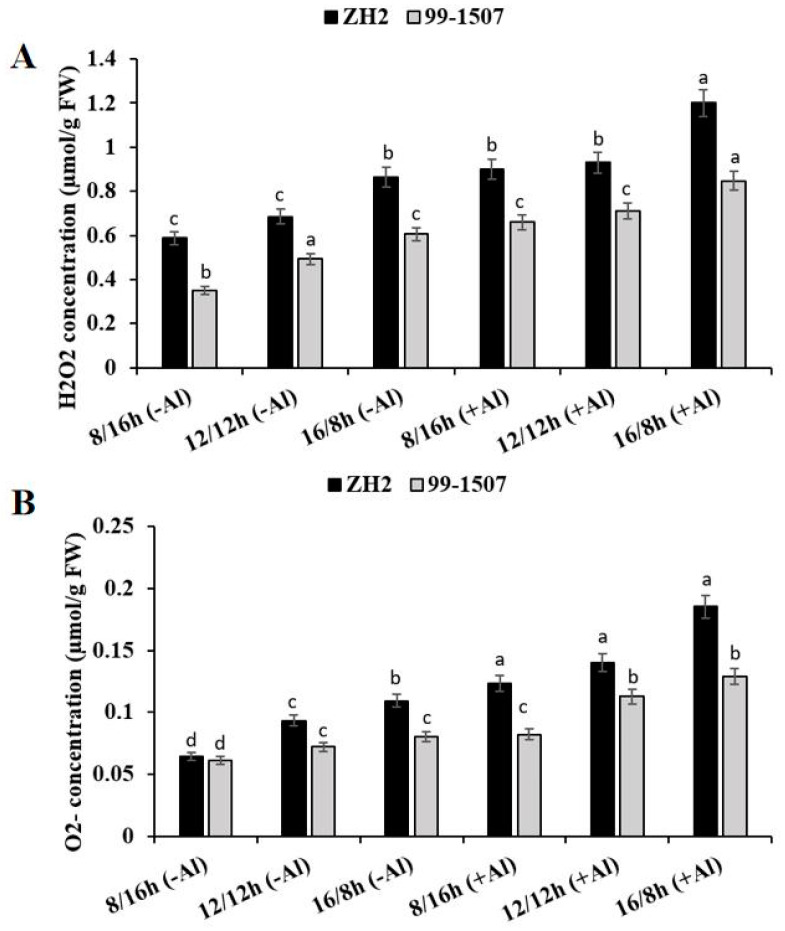
Effect of photoperiodism and Al stress on the production of ROS. (**A**) H_2_O_2_ production; (**B**) O_2_^.−^ production. The experiment was carried out in triplicate to ensure significant results. The data are presented in mean ± standard deviation. Different letters (a–d) assigned to the error bar represent different levels of significance. The results were significant at *p* < 0.05 with different letters.

To detect the levels of ROS production in peanut, H_2_O_2_ and O_2_^.−^ content was analyzed (Figure 3A,B). Root tips were treated under photoperiod alone or the interaction of photoperiod with Al stress. The results show that compared to ND, without Al treatment, the levels of H_2_O_2_ decreased by 14.3% but increased by 26.16% in ZH2 and decreased by 28.84% but increased by 22.75% in 99-1507 under SD and LD, respectively. With Al treatment, H_2_O_2_ increased by 31.53, 35.73, and 75.32% in ZH2 and increased by 33.87, 44.15, and 72% in 99-1507 under SD, ND, and LD, respectively, compared to ND with no Al treatment. The result of O_2_^.−^ content is shown in the Figure 3B. O_2_^.−^ content without Al, compared to ND, decreased by 31.15% but increased by 17.02% in ZH2 and decreased by 14.84% but increased by 10.95% in 99-1507 under SD and LD, respectively. With Al treatment, O_2_^.−^ increased by 32.76, 50.1, and 98.07% in ZH2 and increased by 13.73, 56.17, and 78.91% in 99-1507 under SD, ND, and LD, respectively, compared to ND with no Al. There was a significant effect of photoperiod stress alone or together with Al stress on ROS production, but the level was higher in ZH2 than in 99-1507 and higher under LD than under SD (Figure 3A,B). 

**Figure 4:**
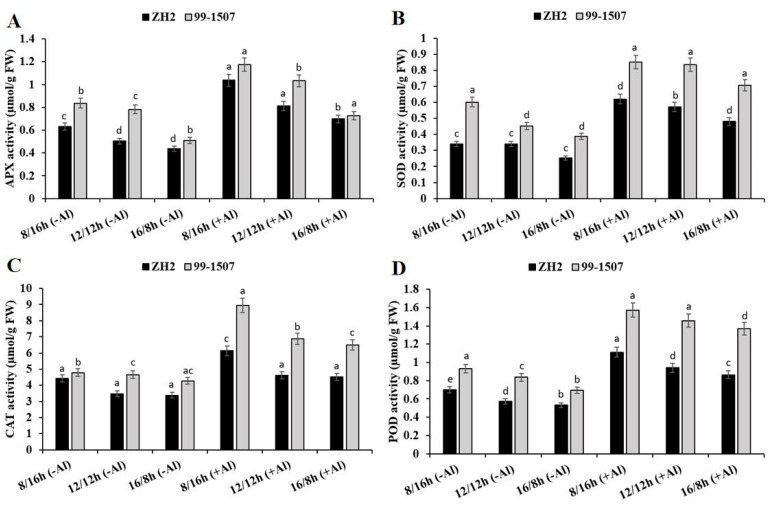
Effect of photoperiodism and Al stress on antioxidant enzyme activities. (**A**) APX activity, (**B**) SOD activity, (**C**) CAT activity, and (**D**) POD activity. The experiment was carried out in triplicate to ensure significant results. The data are presented in mean ± standard deviation. All the samples were used as fresh weight. Different letters (a–d) assigned to the error bar represent different levels of significance. The results were significant at *p* < 0.05 with different letters.

There was a significant effect of photoperiod or photoperiod interacted with Al stress on antioxidant enzyme activities in ZH2 and 99-1507 cultivars of peanut (Figure 4). APX (Figure 4A), SOD (Figure 4B), CAT (Figure 4C), and POD (Figure 4D) activities were induced under photoperiod or photoperiod–Al stress interaction. Without Al treatment, compared to ND, APX increased by 25.47% but decreased by 13.31% in ZH2 and increased by 6.95% but decreased by 34.8% in 99-1507 under SD and LD, respectively. Compared to ND with no Al, APX increased by 105.91, 61.47, and 61.47% in ZH2 and increased by 50.32, 31.91, and 6.93% in 99-1507 under SD, ND, and LD, respectively, under Al stress. Compared to ND, without Al stress, SOD activity increased by 0.05% but decreased by 25.8% in ZH2 and increased by 33.13% but decreased by 14.19% in 99-1507 under SD and LD, respectively. However, under Al stress, SOD activity increased by 82.24, 67.63, and 41.09% in ZH2 and increased by 88.48, 85.16, and 56.36% in 99-1507 under SD, ND, and LD, respectively. The highest CAT activity was under SD as opposed to ND or LD (Figure 4C). Without Al treatment, compared to ND, CAT activity increased by 26.83% but decreased by 2.83% in ZH2 and increased by 2.89% but decreased by 8.1% in 99-1507 under SD and LD, respectively. With Al treatment, compared to ND with no Al, CAT activity increased by 76.3, 32.52, and 30.19% in ZH2 and increased by 92.34, 47.82, and 39.76% in 99-1507 under SD, ND, and LD, respectively. We also detected POD activity and found that, without Al treatment, POD activity increased by 21.82% but decreased by 7.86% in ZH2 and increased by 11.15% but decreased by 17.02% in 99-1507, while with Al treatment, POD activity increased by 92.95, 63.1, and 49.92% in ZH2 and increased by 87.79, 73.82, and 63.56% in 99-1507 under SD, ND, and LD, respectively. Compared to photoperiod alone, Al stress significantly increased APX, SOD, CAT, and POD activities (Figure 4).

### 2.5. Effect of Photoperiod on AhLHY and AhSTS Gene Expression under Al Stress 

**Figure 5:**
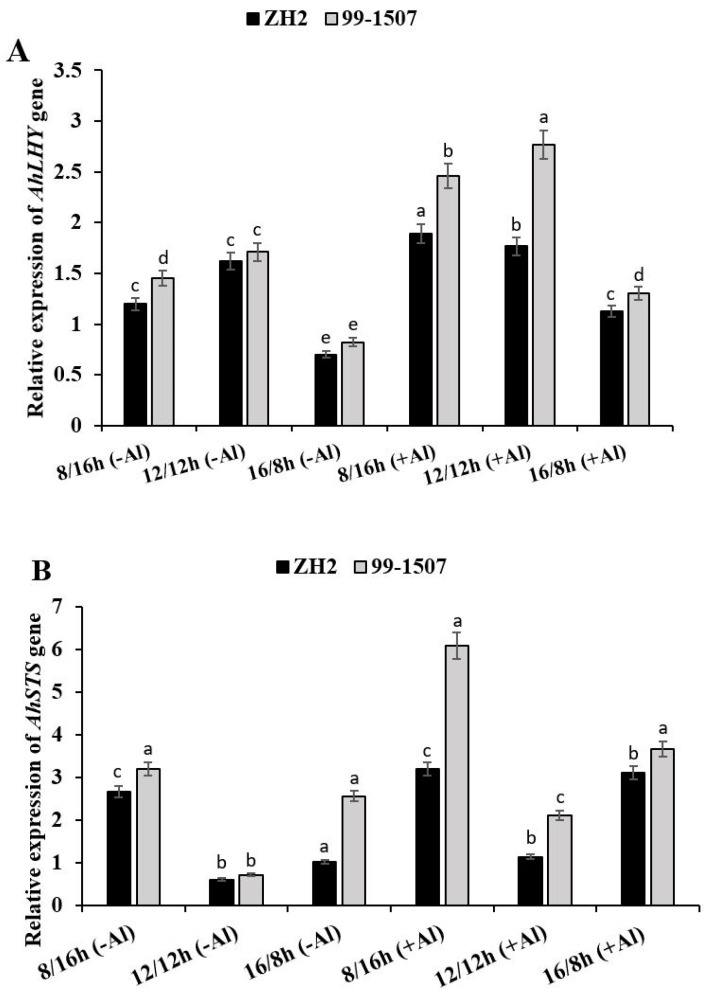
Relative expression of *AhLHY* (**A**) and *AhSTS* (**B**) genes in peanut treated with Al or without Al under three photoperiods. The data in the figure were obtained from three treatments, and analysis of variance (ANOVA) was performed; *p* < 0.05 indicated that the gene differential expression is significant among the three treatments; *n* = 3; Different letters assigned to the error bar represent different levels of significance, error bar is presented as mean ± standard deviation; reference gene was *AhActin*.

To obtain insight into the potential functional roles of photoperiod in Al stress, two genes, *AhLHY* (AH02G04520.1) and *AhSTS* (AH04G08660.1), were selected based on the transcriptome (PRJ-NA525247) in NCBI to detect their expression levels under Al stress through qRT-PCR (Figure 5). Both the *AhLHY* and *AhSTS* genes were up-regulated under photoperiod alone or interaction of photoperiod with Al treatment in ZH2 and 99-1507 (Figure 5A, B). Photoperiod positively regulated expressed *AhLHY* (Figure 5A), and the expression highly shifted when photoperiod interacted with Al treatment. Without Al treatment, compared to ND, *AhLHY* expression level decreased by 25.92 and 56.7% in ZH2 and decreased by 38.87 and 36.38% in 99-1507 under short day (SD, 8/16 h) and long day (LD, 16/8 h), respectively. To analyze the level of expression before and after Al treatment, we compared ND without Al to the treatments under Al stress. *AhLHY* expression increased by 9.25 and 51.72% in ZH2 and increased by 62.04 and 10.52% in 99-1507 under ND and SD, respectively. However, it decreased by 30.49% in ZH2 and 23.85% in 99-1507 under LD. The highest expression level was observed in 99-1507 with Al treatment under ND, while the lowest was observed in ZH2 without Al treatment under LD (Figure 5A). *AhSTS* expression under ND was lower than it was under SD or LD, but it was also higher under SD than it was under LD or ND without Al stress (Figure 5B). On the other side, when treated with Al, compared to ND with no Al, *AhSTS* expression increased dramatically by 5.33, 1.88, and 5.16 times in ZH2 and increased by 8.57, 2.97, and 5.16 times in 99-1507 under SD, ND, and LD, respectively. The highest expression level of *AhLHY* expression was observed under ND in 99-1507 and the lowest level was ZH2 under LD. Contrastingly, the highest expression level of *AhSTS* was observed in 99-1507 under SD, and the lowest level was in ZH2 under ND. Compared to photoperiod alone, interaction of photoperiod with Al treatment significantly increased the expression of *AhLHY* and *AhSTS* (Figure 5B).

## 3. Discussion

### 3.1. Effects of Photoperiods on Root Elongation and Cell Death under Al Stress

Environmental changes such as seasonal variation in photoperiod can modulate circadian rhythms, allowing organisms to adjust to the time of the year [50]. Photoperiod stress causes the induction of numerous stress responsive genes, which are indicators of oxidative stress, during the night following the extended light period. Light itself acts as a stressor and, in addition, regulates the outcome of the Al stress response. Our results show that photoperiod influenced root growth. Photoperiod stress alone affected root growth in a way that compared to ND, and root length under SD was higher than under LD (Figure 1A). However, this contradicted the study in radish (long-day plant), which stated that long photoperiod enhanced the formation of radish root [51], but it may be consistent with the study on potato, which revealed that tubers below ground were formed under SD [52]. There was a significant influence of photoperiod and Al stress on root growth; root growth was highly inhibited under LD and SD compared to ND. Numerous reports have discussed the effect of Al stress and revealed that plants exposed to Al stress result in the inhibition of root growth and decrease in crop production [53]. In the present study, we showed that different photoperiods together with Al stress affected root growth and inhibited the growth of root and induced cell death in peanut. Though photoperiodic change regulates various processes in plants, it can also induce numerous stresses [54]. Without Al stress, we observed more dead cells under LD and SD compared to ND (Figure 2). Though there was a significant effect of photoperiod on Al content in root tips and CW under Al stress, there was no significant effect without Al treatment (Figure 1C,D). A recent study revealed that during the night after the prolongation of the light period, stress and cell death marker genes were induced in Arabidopsis [55]. Our results show that root growth was highly inhibited in ZH2 under LD rather than SD, and cell death was also highly induced under LD rather than SD in ZH2. This reveals that peanut is highly sensitive to Al stress under LD.

### 3.2. Effect of Photoperiod and Al Stress on MDA, PME, and LOX Activity

Photoperiod affected the level of lipid peroxidation; hence, MDA formation was strongly affected by the photoperiodic changes [43]. Our result shows that, compared to ND, both photoperiod alone and photoperiod together with Al stress influenced MDA content in a way that MDA level was higher under LD than under SD (Figure 2A), and this difference was highly observed under Al stress. The highest MDA content was higher in ZH2 than 99-1507. It shows that LD and Al stress lead to membrane lipid peroxidation in peanut, which is more serous in Al-sensitive cultivar and LD. A recent study reported that MDA content was higher under LD in leaves of *Pfaffia* *glomerata*, leading to higher levels of membrane lipid peroxidation and signaling photooxidative damage [56]. In peanut, increased MDA content is an indicator of membrane lipid peroxidation and abnormal root growth [57]. 

LOX initiates subsequent biological reactions and activates cellular signaling mechanisms through specific cell surface receptors [58]. LOX activity is involved in catalyzing the formation of H_2_O_2_ derivatives and activating the lipid peroxidation of membranes. In our study, we found that LOX activity was significantly affected by photoperiod, but this activity level was higher under Al stress (Figure 2C). Compared to ND, LOX was higher under LD than under SD in both ZH2 and 99-1507, but it was also higher in ZH2 than 99-1507. It has been reported that, in potato, LOX activity in morning glory was greatly enhanced and then declined after switching from the light to the dark condition, while the activity did not vary when switching from the dark to the light condition [59]. 

PME activity reduces pectin methylation [60]. The lower the level of pectin methylation in the cell wall, the greater the Al accumulation was in the cell wall and root tips [61]. In this study, we found that photoperiod influenced PME activity, and its activity level was lower under SD than under LD in both ZH2 and 99-1507 (Figure 2B); this influence was higher under Al stress than photoperiod alone. This increase in PME activity level in the presence of Al stress was as the response to Al stress [57]. In the present study, Al toxicity was higher in ZH2 under LD. This was consistent with the higher Al accumulation measured in ZH2 and LD, demonstrating that the increased PME activity induced by Al and LD accelerated Al toxicity. 

Our results consistently suggest that MDA content, PME activity, and LOX activity are directly connected with photoperiod and Al stress.

### 3.3. Circadian Rhythm Regulates Al-Induced PCD by Controlling ROS Production and Antioxidant Enzyme Activity

Light is an important source of energy and a developmental signal for plants, but it can also cause stress to plants and modulates responses to stress and PCD; excess and fluctuating light result in photoinhibition and ROS accumulation [62]. ROS production plays a critical role in plant development, response to abiotic stresses and immune responses. In the present study, we found that photoperiod could influence ROS production itself but also influence ROS induced by Al stress (Figure 3). Compared to ND with no Al treatment, H_2_O_2_ and O_2_^.−^ production levels were significantly higher under LD than under SD and ND in both ZH2 and 99-1507, but they were greater in ZH2 than in 99-1507 under Al stress (Figure 3A, B). In SD-entrained Arabidopsis, shorter prolongation of the light period causes lower stress levels, is perceived as not harmful and may present a beneficial stress, while higher stress levels by longer prolongations induce a true stress [63]. ROS production in the form of H_2_O_2_ and O_2_^.−^ was significantly affected by photoperiod. However, it was highly affected by photoperiod interacted with Al stress. The lower levels of ROS production in 99-1507 (Al tolerant) under SD rather than LD are evidence that peanut is more resistant to Al stress under SD than under LD (Figure 3A,B). A recent study has revealed that LD species are generally more Al-sensitive than SD species and that the genetic conversion of tomato for the SD growth habit boosts Al tolerance [64]. 

The day-length sensing mechanisms have been identified to be diverged more between LD plants and SD plants than the circadian clock [65]. In the present study, we found that photoperiod and Al stress induced ROS and activate cellular endogenous antioxidant systems to prevent oxidative stress. The presence of ROS induced various antioxidant enzyme activities, such as APX, SOD, CAT, and POD. There is evidence that ROS play a critical role as the signaling molecules throughout the entire cell death pathway [66]. When we treated ZH2 and 99-1507 peanut cultivars under different photoperiods, compared to ND, APX, SOD, CAT, and POD activities were lower under LD than under SD and antioxidant enzyme activities were higher in 99-1507 than in ZH2 (Figure 4). Further analysis under Al stress revealed that antioxidant enzymes were highly activated. Compared to ND with no Al treatment, APX, SOD, CAT, and POD activities were also higher under SD, ND and LD, respectively, in ZH2 and 99-1507 (Figure 4). In rye, CAT was degraded under light, and the degradation was clearly observed from 16 h after the onset of light [67,68].

### 3.4. Effect of Photoperiod and Al Stress on AhLHY and AhSTS Gene Expression

LHY/CCA1 regulates photoperiodic flowering, and it has been found that LHY-defective mutants (lhy-7 and lhy-20) exhibit accelerated flowering under both LD and SD [69]. In this study, we observed a remarkable difference between AhLHY gene expression in ZH2 and 99-1507 under ND, SD, and LD either treated with photoperiod alone or together with Al (Figure 5A). Though the influence of photoperiod on AhLHY expression was significant, it was highly significant under the interaction with Al stress. The highest expression level was in 99-1507 under ND and Al stress, but this expression level was also higher under SD than it was under LD (Figure 5A). LHY, CCA1, and TOC1 constitute the core of the circadian clock [69]. We confirmed that the expression pattern of *AhLHY* under photoperiod and Al stress reveals the role of the circadian clock in Al stress control. A previous study revealed that the integration of abiotic stress response into the circadian system provides control over daily plant metabolism [70].

*Peanut Stilbene synthase (AhSTS)* expression was also influenced by photoperiod, but photoperiod together with Al treatment influenced the expression of *AhSTS* more (Figure 5B). *AhSTS* was highly expressed in 99-1507 and ZH2 under SD rather than LD and ND, but it was higher in 99-1507 than in ZH2. However, we could not find evidence to prove that this photoperiodic influence is similar to the circadian clock. Therefore, further study is needed to investigate the molecular feature of *AhSTS* under circadian rhythm. Plants may be classified as long-day, short-day, or neutral, and their resistance toward the stress is different. Peanut is a short-day crop [71]. Al toxicity was clearly observed in ZH2 under LD rather than under SD, and it was more tolerant in 99-1507 under SD than under LD. Al induced ROS production, antioxidant enzyme activities, and *AhLHY* and *AhSTS* expression under different photoperiods; this proves the role of photoperiodism in the regulation of Al-induced PCD. 

Here, we proved that peanut as a short-day crop is more sensitive to Al stress under LD and more Al-tolerant under SD. Overall, this study reveals that day length plays an important role in determining whether ROS production is enhanced under Al stress. Understanding the physiology of the plant face to Al stress under photoperiod can help to regulate Al stress in plants. However, deeper investigation is needed to truly understand the molecular mechanisms and pathways of circadian clock systems under photoperiod and Al stress in plants. 

## 4. Materials and Methods

### 4.1. Plant Material and Growth Conditions 

Two varieties of peanut, ZH2 (Al-sensitive) and 99-1507 (Al-tolerant), were prescribed as Al-sensitive and Al-resistant, respectively, and were used as plant materials. Plant material and growth condition preparation was conducted following the method of [72] with little modifications. In short, the seeds of peanut were placed into wet perlite sand for 3–4 days at 26 ± 2 °C to induce germination. After germination, the seedlings were 2–3 cm long and were placed in Hoagland nutrient solution for 4 days (d). After 4 d, the seedlings had four leaves and were pretreated with 0.1µM CaCl_2_ solution at pH 4.2 for 24 h; then, the seedlings were treated with 100 µM AlCl_3_ for 24 h at three different lighting periods (8/16, 12/12, and 16/8 h light–dark periods). All the treatments were conducted in a controlled environment with 26 ± 2 °C, 70% relative humidity (RH), and light intensity 2000 lux. Al was washed off the root surface prior to the analysis to avoid bias, which may result in the influence of external Al.

### 4.2. Relative Root Growth, Evan’s Blue Staining, and Cell Death Assay

To measure root elongation, Al-treated peanut root tips (main roots) under the circadian rhythms were cut. The data were presented by a histogram chart as relative root elongation. The main root tips were stained with Evan’s blue, and the picture was taken by a Canon scanner (DR-S150). For cell death assay, root tips (approximately 1 cm) were stained with 0.5% (*w*/*v*) Evan’s blue for 15 min and rinsed in distilled water for 30 min. Stained root tips were immersed in a centrifuge tube containing 4 mL N, N-dimethylformamide for 1 h; then, a solution containing Evan’s blue dye, which had leached from dead cells, was measured at 600 nm with spectrophotometer U*V*/*V*IS (specord plus 50, Analytik Jena, Konrad-Zuse-Strasse, Germany).

### 4.3. Al Content in Root Tips and Cell Wall

To assay the total Al content in the root tips, fresh root tips were cut (approximately 1 cm), rinsed in 1 mL of 2 M HCl, and then incubated at 25 °C for 24 h with occasional shaking to ensure that Al was released from the root tips. The upper solution was collected and used to measure the total Al content in root tips. To determine Al content in the CW, root tips were collected, frozen in liquid nitrogen, grounded with mortar and pestle, and then homogenized in 7 mL 75% ethanol and left on ice for 20 min. The homogenized samples were centrifuged at 13,000× *g* for 15 min, and the supernatant was discarded. The pellets were washed with precooled (4 °C) acetone followed by methanol:chloroform (1:1), and then with methanol. After washing, the pellets were dried in an oven for 12 h at 60 °C and suspended in 1 ml 2 M HCl for 24 h at room temperature (RT) with occasional shaking. Al content in the root tips and CW were measured by U*V*/*V*IS spectrophotometer (specord plus 50, Germany) at 600 nm. 

### 4.4. Lipid Peroxidation and PME Activity Assay

To analyze the lipid peroxidation (LPO) levels, the malondialdehyde (MDA) content was assayed following the method of [72] with little modifications. In brief, approximately 0.2 g fresh root tips were collected and stored in liquid nitrogen at −80 °C. Thereafter, the root tips were grounded and homogenized in 10 mL 10% trichloroacetic acid (TCA). The homogenate was centrifuged at 13,000× *g* for 10 min. We aliquoted 2 mL of the supernatant mixed with 2 mL 0.6% (*w*/*v*) thiorbarbituric acid in 10% TCA, and incubated in the water bath at 95 °C for 15 min. The ice-cooled mixture was centrifuged at 13,000× *g* for 10 min, and the supernatant was measured at 450, 532, and 600 nm. The MDA content ([C]) was calculated using the following formula:

$$\mathrm{MDA}\;\left[C\right]\;\left(\mathrm{nM}\right)=6.45\times \left(A532-A600\right)-0.56\times A450$$

To determine the pectin methylesterase (PME) activity, 0.2 g of the root tips was grounded and homogenized with 2 mL 1 M sodium chloride (NaCl) containing 1% (*w*/*v*) polyvinylpyrrolidone (PVP) then centrifuged at 13,000× *g* for 20 min, 4 °C. The enzyme activity was analyzed by mixing 1 ml 0.5% (*w*/*v*) pectin (pH 7.5), 0.4 mL 0.01% (*w*/*v*) bromothymol blue (pH 7.5), 1.55 mL distilled water (pH 7.5), and 50 µL of the root tip extract; then, the mixture was measured with a spectrophotometer at 620 nm. 

### 4.5. ROS (H_2_O_2_ and O_2_^.−^) Content

The H_2_O_2_ and O_2_^.−^ content in the root tips was detected following the method of [73]. Briefly, the root tips of peanut (0.5 g) were ground with mortar and pestle in liquid nitrogen, then homogenized with 3 mL of cold acetone and centrifuged at 5000× *g* at 4 °C for 10 min. A total of 1 ml of the supernatant was mixed with 0.1 mL of 5% (*w*/*v*) titanic sulfonate (Ti(SO_4_)_2_) and 0.1 mL 25% ammonia, and was centrifuged at 3000× *g* for 10 min at 4 °C; the pellet was resuspended in 4 mL of 2 N sulfuric acid (H2SO4). H_2_O_2_ content was spectrophotometrically determined at 415 nm. 

O_2_^.−^ content was measured as described by [73]. Root tips (0.5 g) were homogenized in 2 mL of 65 mM phosphate buffer (pH 7.8), then centrifuged at 5000× *g* for 10 min at 4 °C. The aliquot of 1 mL from the supernatant was mixed with 0.9 mL of 65 mM phosphate buffer (pH 7.8) and 0.1 mL of 10 mM hydroxylamine hydrochloride (HONH_2_·HCl), then the mixture was incubated at 25 °C for 20 min. A total of 1 ml of the mixture was extracted and added to 1 mL of 17 mM anhydrous aminobenzene sulfonic acid (H_3_NC_6_H_4_SO_3_), as well as 1 mL of 17 mM 1-naphthylamine (C_10_H_9_N). The mixture was incubated at 25 °C for 20 min, then 3 ml of n-butanol was added. The O_2_^.−^ content was measured at 530 nm.

### 4.6. Antioxidant Enzyme Activity

To determine the antioxidant enzyme activity, root tips (0.2 g) were ground with mortar and pestle in liquid nitrogen and then homogenized with 50 mM sodium phosphate buffer (pH 7.8) containing 1 mM ethylenediaminetetraacetic acid (EDTA), 2% (*w*/*v*) PVP, 1 mM phenylmethanesulfonyl fluoride (PMSF), 1 mM dithiothreitol (DTT), and 0.05% (*v*/*v*) Triton X-100 and centrifuged the homogenate at 13,000× *g* for 15 min at 4 °C. Lipoxygenase (LOX) activity was assayed following the method of [72]. In total, 50 µL of the supernatant was extracted and mixed with 2.75 mL potassium phosphate buffer (pH 6.5), 0.2 ml 7.5 mM linoleic acid containing 0.25% (*v*/*v*) Tween 20, and then measured at 234 nm. CAT, APX, and SOD activities were assayed following the method of [73]. A total of 0.1 ml of the root tips was extracted with 1.9 ml 0.5 M phosphate buffer pH 7.0, and 1 ml 0.5 mM 30% H_2_O_2_ solution and CAT activity was spectrophotometrically measured as H_2_O_2_ decomposition at 240 nm. To detect APX activity, the root tip extract was mixed with 0.25 mM ascorbic acid, 0.5 mM H_2_O_2_ and measured at 290 nm. SOD activity was determined, as the root tips were homogenized in 1 ml cold 100 mM potassium phosphate buffer pH 7.8 containing 0.1 mM EDTA, 1% PVP, and 0.5% *v*/*v* triton x-100, and it was measured at 560 nm, as enzyme amount required us to inhibit the reduction of 50% of NBT at 560 nm. 

POD activity was assayed following the “standard operating procedures” of scientific engineering response and analytical services (SERAS, SOP 2035, page: 1–7, date: 11/28/94). Frozen root tips (1 g) were ground using mortar and pestle with liquid nitrogen; then, they were homogenized into ice-cold 0.5 M calcium chloride solution (CaCl_2_). After the homogenization, the solution was centrifuged at 1000× *g* for 8 min. The supernatant was extracted into a 10 mL centrifuge tube and kept on ice. The pellet was resuspended with 2.5 mL 0.5 M CaCl2 and centrifuged again (repeated twice). Before POD assay, buffer solutions (A and B) were brought to 25 °C with a water bath. We mixed 1.4 mL of solution A(Phenol and 4-aminoantipyrene), 1.5 mL of solution B (0.5 mL of 30% H_2_O_2_ and MES or HEPES buffer solution, and the volume was brought to 50 mL to make a 3% H_2_O_2_ solution with 0.01 M final buffer concentration), and 200 µL roots extract. Then, we measured the absorbance at 510 nm. All activities were measured with a spectrophotometer (Specord 50 plus U*V*/*V*IS, Germany).

### 4.7. Quantitative Real-Time Polymerase Chain Reaction (qRT-PCR) Analysis 

Total RNA was extracted using Eastep^®^ Super Total RNA Extraction KitRNeasy LS1040 (Promega, Shanghai, China) following the manufacturer’s instructions. The quality of RNA was proved by the ThermoFisher Scientific NanoDrop 2000c, Germany (RNA concentration 1.9–2.2 µg was used). cDNA was synthesized through RNA reverse transcription using PrimeScriptTM RT reagent Kit with gDNA eraser (Perfect Real Time) Cat#RR047A, Takara bio, China. qRT-PCR was performed with CF × 96 TM Real-Time System, Bio-Rad Laboratories, Hong Kong, using SYBR Green qPCR super mix. The actin was used as the normalization control. The following primers were used:


**Gene Name**

**Gene ID**

**Primer**
AhLHY-forwardAH02G04520.15′-ATTGACTCTAGTAATCGTCGTA-3′AhLHY-reverse5′-CTTTGTGGCAACACCTCT-3′AhSTS-forwardAH04G08660.15′-CCCAAGCGTCAAGAGGTA-3′AhSTS-reverse5′-TTGCCCACAAGACTATCCA-3′Actin-forwardAH03G02610.15′-ACCTTCTACAACGAGCTTCGTGTG-3′Actin-reverse5′-GAAAGAACAGCCTGAATGGCAAC-3′

### 4.8. Statistical Analysis 

The experiments were independently replicated three times and their mean values were subjected to sata processing and statistical analysis with Excel 2007 and SPSS 12.0 (SPSS software Inc., Chicago, IL, USA). Statistical analysis was performed with Student’s paired *t*-test to test the differences between groups. The data are presented as mean ± standard deviation (SD). *p*-value ≤ 0.05 was considered statistically significant.

## 5. Conclusions

Different light–dark conditions showed different levels of Al toxicity, which could reveal the influence of photoperiod in the regulation of Al stress. The induction of ROS by Al stress led to the deterioration of the cell wall, the release of free radicals (H_2_O_2_ and O_2_^.−^), and the acceleration of lipid peroxidation; increased MDA concentration; accelerated PME activity; and positively induced the expression of LHY and STS genes. Prolongation of the light period resulted in photoperiod stress, and it has an important influence on Al stress and tolerance. Peanut showed a strong resistance to Al toxicity under SD, moderate under ND, and low under LD. The molecular mechanism and pathways of *AhLHY* and *AhSTS* in Al tolerance under the circadian rhythms should be studied next, including the metabolic engineering of stilbene biosynthesis as a strategy to directly demonstrate the role of this phytoalexin in plant stress resistance.
